# Median nerve in the distal forearm: A Cadaveric Morphometric Study

**DOI:** 10.1097/MD.0000000000048308

**Published:** 2026-04-10

**Authors:** Ceren Uğuz Gençer, Hüseyin Aydoğmuş, Mustafa Deniz Yörük, Zeynep Nisa Karakoyun, Mehmet İlkay Koşar, Hasan Tetiker

**Affiliations:** a Department of Anatomy, Faculty of Medicine, Muğla Sitki Koçman University, Muğla, Turkey; b Department of Physical Medicine and Rehabilitation, Faculty of Medicine, Muğla Sitki Koçman University, Muğla, Turkey.

**Keywords:** anatomy, carpal tunnel syndrome, distal forearm, median nerve, morphometry

## Abstract

The median nerve (MN) is a main peripheral nerve that includes superficial motor and sensory fibers in the distal forearm. The proximity of the MN to the forearm muscle tendons and neurovascular structures enhances its clinical significance. The safety of invasive procedures in this area relies on a thorough understanding of the nerve morphometric characteristics. However, commonly used superficial anatomical landmarks may not provide the same reliability for every individual. This study aimed to precisely identify the morphometric features of the MN in the distal forearm by using fixed bony landmarks and to evaluate its positional relationships with nearby anatomical structures. Measurements of the length, width, surface area, and distances from the MN to the ulnar styloid process, radial styloid process, flexor carpi radialis tendon, and ulnar nerve were measured by dissecting the forearms of 31 formalin-fixed cadavers. Differences in gender and side were evaluated, along with the connections between wrist width and various morphometric parameters. There was no statistically significant difference between genders or sides. Without regard to sex or side, the mean distance from the MN to the radial styloid process was 20.97 ± 3.02 mm, to the ulnar styloid process was 19.44 ± 4.83 mm, and to the flexor carpi radialis tendon was 3.83 ± 0.96 mm. The mean surface area of the nerve was 1.49 ± 0.63 cm^2^. Median nerve-ulnar nerve distance was 12.09 ± 3.77 mm, and it remained within a narrow range. Furthermore, a positive correlation was observed between wrist width and both nerve length and surface area. The morphometric features of the MN in the distal forearm consistently align with bone landmarks, making these structures a more reliable guide than variable superficial tendon references. These data will contribute scientific knowledge to improve the safety of interventional methods, particularly in distal forearm and hand surgery. However, as these findings are based on cadaveric specimens, their direct clinical applicability should be interpreted with caution and warrants further validation in living subjects.

## 1. Introduction

The median nerve (MN) is a mixed nerve comprising sensory and motor fibers and is critical to hand function. Besides controlling many muscles in the forearm and hand, it also transports the palmar fascia of the hand.^[1]^

MN, which runs without branching on the anterior side of the arm, passes between the 2 heads of the pronator teres muscle and enters the forearm. In the forearm, the flexor digitorum runs deep to the superficialis and superficially to the flexor digitorum profundus; more distally, the flexor digitorum becomes visible lateral to the superficialis tendon. During its course, it moves towards the hand along the medial side of the flexor carpi radialis (FCR) tendon and, if present, along the lateral or deep side of the palmaris longus (PL) tendon. The most superficial position before entering the carpal tunnel is visible here, and at this point, the PL tendon can be palpated on the lateral side. The tendons of the FCR and PL can serve as landmarks to locate the nerve. However, only a portion of the MN in the forearm is protected by the PL tendon; the remaining part lies superficially and is susceptible to traumatic and iatrogenic injuries. Its superficial course, which is not well protected by muscle tissue, makes this area susceptible to trauma. The nerve may be injured in wrist fractures and dislocations either by pressure from the broken ends, direct contusion, or, rarely, complete tearing.^[2–4]^ The risk of injury rises even more when the PL muscle is missing. Furthermore, penetrating injuries to the wrist can result in complete or partial severance of the MN. MN injuries are quite common during wrist lacerations, forearm fractures, and regional surgeries.^[5]^ Additionally, MN injury is a complication of carpal tunnel injections, and the actual incidence is unknown.^[6]^ Depending on the technique employed for corticosteroid injections in patients with carpal tunnel syndrome(CTS), there is a risk of iatrogenic injury to the MN, the ulnar nerve (UN), or the radial artery.^[7]^ Various injection techniques are proposed in the literature, but consensus has not yet been reached.^[8]^

It has been reported that the MN is the most frequently affected in iatrogenic peripheral nerve injuries.^[9]^ Such injuries may result in long-term or permanent motor and sensory impairment. The proximity of a nerve to the injection site is one of the most critical factors in determining the severity of potential damage.^[10]^ Therefore, understanding peripheral nerve anatomy and choosing appropriate invasive techniques are essential for preventing MN injuries.

Although numerous studies in the literature address the cross-sectional area of the MN in the carpal tunnel, its branching patterns, and anatomical variations, there is limited data on the area where the flexor digitorum superficialis (FDS) muscle becomes superficially lateral in the forearm. However, this region is vulnerable to trauma and is a commonly encountered area during surgical procedures. Therefore, a detailed examination of the MN path in this section is clinically important.

In this study, we aimed to analyze the morphometric characteristics (width, area, and length) of the MN in the distal forearm. We also examined its relationships, as well as its position and the distances relative to the adjacent anatomical structures, including palpable bony landmarks, tendons, and neighboring nerves. By describing the distal course of the MN in a cadaveric model, this study seeks to provide anatomical reference data that may assist in identifying the relative location of the nerve, rather than defining definitive or clinically applicable safe zones.

## 2. Methods

This is a descriptive cross-sectional observational study involving formalin-fixed human cadavers, conducted with approval from the local ethics committee. The average age of the cadavers was 71.06 ± 16.27 years (minimum 48, maximum 101). Due to the nature of cadaver procurement, the sample size was determined under practical limitations. In cadaver-based studies evaluating nerve and wrist morphometry in the distal forearm, sample sizes are generally limited. The literature reports analyses by Ajayi et al^[5]^ with 14 female and 16 male samples, Vavricka et al^[4]^ with 15 cadavers, McCann et al^[2]^ with 10 forearms, and Brooks et al^[8]^ with 7 forearms. In this study, 31 forearms that were dissected (5 females, 12 males) from all cadavers that had been found suitable for the study in the anatomy laboratory to date were included. Only 1 forearm was excluded from the study because it was not considered reliable in terms of measurements due to deformity. Of the forearms included in the study, 14 were bilateral, comprising 4 female and 10 male specimens. Macroscopically, none of the specimens showed any signs of pathology. Before beginning the dissection, the forearm was positioned in supination.

**Figure 1.**
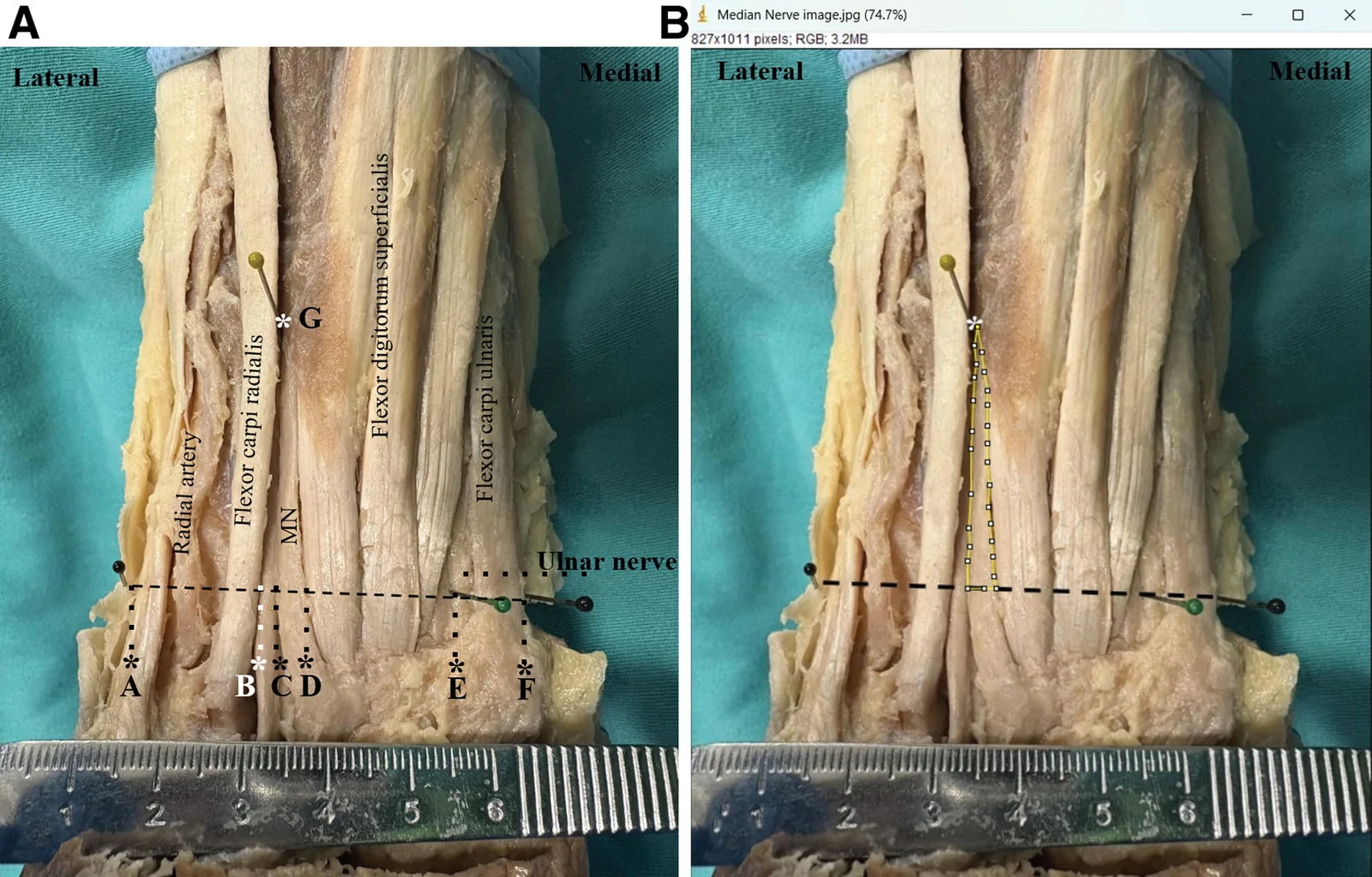
Measurement of morphometric parameters using the ImageJ software. Anterior view of the right distal forearm. (A) A to F: bistyloid line length (BL); G-midpoint of C to D:median nerve length (MNL); C to D: median nerve width (MNW); B to C: distance from median nerve (lateral edge) to FCR tendon (MN–FCR); D to E: distance from median nerve (medial edge) to ulnar nerve, (MN–UN); D to F: distance from median nerve (medial edge) to ulnar styloid process (MN–USP); C to A: distance from median nerve (lateral edge) to radial styloid process (MN–RSP). (B) Yellow line: surface area of the median nerve (MNA). FCR = flexor carpi radialis muscle.

On the anterior surface of the cadaver forearms, the radius styloid process (RSP) and ulna styloid process (USP) bones were identified, with dissection carried out in the neutral position. After the fascias were separated in the forearms, where the skin and subcutaneous adipose tissue had been previously removed, the muscles of FCR, FDS, and PL were identified. Then, the MN was identified and traced to the point where it left the FDS. After exposing the nerve, its surrounding features and morphometric data were recorded. All structures were fixed and photographed by marking them with colored needles. The photos were captured in a computer environment and transferred to ImageJ, an open-source software (ImageJ 1.54g, National Institutes of Health, Bethesda) for image visualization, processing, and analysis. Calibration was performed using the ruler shown in the image, and all measurements were taken from the calibrated images. The following morphometric measurements were taken twice along the bistyloid line (BL) in millimeters (mm) by the same observer using a digital caliper (D&W, China; model dw1kds15). The measurements were averaged, and the resulting values were recorded and entered into the computer system (Fig. 1).

- **BL length:** Linear distance between the radial styloid process and the USP
- **MN length:** The length of the MN is measured from where it becomes superficial on the lateral edge of the FDS to where it crosses the BL
- **MN width:** The width of the MN at the intersection of the BL
- **MN–FCR:** Distance from the lateral edge of the MN to the FCR tendon
- **MN–UN:** Distance between the medial edge of the MN and the UN
- **MN–USP**: Distance between the medial edge of the MN and the USP
- **MN–RSP:** Distance between the lateral edge of the MN and the RSP
- **MN area:** Area of the superficial part proximal to the BL

Statistical analyses were performed using SPSS (Version 27.00; IBM Corp., Armonk) software. The appropriateness of the variables for normal distribution was visually evaluated using histograms and probability (*Q*–*Q*) plots, as well as analytically tested with Kolmogorov–Smirnov and Shapiro–Wilk tests. Descriptive statistics were presented as mean ± standard deviation and median (minimum–maximum) values for continuous variables. The difference between genders in normally distributed variables was compared using Student *t* test, and in nonnormally distributed variables using the Mann–Whitney *U* test. When appropriate for bilateral measurements obtained from the same cadaver, paired comparisons (paired-samples *t* test or Wilcoxon signed-rank test) were used. Relationships between quantitative variables were evaluated using Pearson correlation for normally distributed data and Spearman correlation for nonnormally distributed data.

In the study, 3 different ratios were also established to evaluate the proportional relationships between anatomical structures: bistyloid width/MN width, MN area/MN width, and the ratio of the MN distance to the radial styloid to its distance to the ulnar styloid. The differences in these ratios by gender and side were assessed using Student *t* test, Mann–Whitney *U* test, or Wilcoxon signed-rank test, depending on the distribution.

The significance level was set at *P* < .05 for all statistical analyses.

## 3. Results

The study examined 31 forearms in total, including 14 bilateral cases from 9 female and 22 male cadavers. No significant difference was observed in the comparison of the MN parameters in the forearm between male and female groups (*P* > .05, Table 1).

**Table 1:** Sex-based distribution of morphometric parameters of the median nerve and adjacent structures at the distal forearm.

	Total (n = 31)	Female (n = 9)	Male (n = 22)	*P*
BL (mm)	48.15 ± 4.28	47.18 ± 2.62 95% CI: 45.16–49.19	48.55 ± 4.80 95% CI: 46.43–50.68	.316*
	47.6 (39.8–57.85)	47.1 (41.50–51.60)	47.85 (39.8–57.85)	
MNL (mm)	35.49 ± 9.71	32.77 ± 5.22 95% CI: 28.76–36.78	36.60 ± 10.95 95% CI: 31.75–41.46	.199*
	34.5 (21.3–62.5)	33.90 (25.10–39.20)	36.71 (21.30–62.50)	
MNW (mm)	7.65 ± 2.16	8.68 ± 2.70 95% CI: 6.61–10.76	7.22 ± 1.80 95% CI: 6.42–8.02	.157†
	7.90 (3.2–14.9)	8.30 (4.80–14.90)	7.35 (3.20–10.40)	
MN–FCR (mm)	3.83 ± 0.96	3.87 ± 0.85 95% CI: 3.21–4.52	3.81 ± 1.02 95% CI: 3.36–4.26	.887*
	3.9 (2.3–6)	3.60 (2.50–5.20)	3.90 (2.30–6.00)	
MN–UN (mm)	12.09 ± 3.77	10.33 ± 2.56 95% CI: 8.36–12.30	12.81 ± 3.99 95% CI: 11.04–14.57	.097*
	11.2 (5.77–22.2)	11.20 (5.77–14.20)	11.50 (7.30–22.20)	
MN–USP (mm)	19.44 ± 4.83	17.44 ± 3.76 95% CI: 14.55–20.32	20.26 ± 5.05 95% CI: 18.02–22.50	.142*
	19.65 (10.9–29.23)	17.67 (10.90–22.85)	20.18 (11.15–29.23)	
MN–RSP (mm)	20.97 ± 3.02	21.22 ± 3.15 95% CI: 18.80–23.64	20.87 ± 3.04 95% CI: 19.53–22.22	.780*
	21.0 (13.55–26.15)	20.70 (16.45–25.81)	21.44 (13.55–26.15)	
MNA (cm^2^)	1.49 ± 0.63	1.63 ± 0.61 95% CI: 1.16–2.10	1.43 ± 0.64 95% CI: 1.41–1.71	.286†
	1.30 (0.66–3.11)	1.59 (1.03–2.77)	1.26 (0.66–3.11)	

Descriptive statistics are presented as median (minimum–maximum).

Confidence intervals (95% CI) are provided for mean values where applicable.

BL = bistyloid line length, MNA = median nerve area, MN–FCR = distance from median nerve (lateral edge) to flexor carpi radialis muscle tendon, MNL = median nerve length, MN–RSP = Distance from median nerve (lateral edge) to radial styloid process, MN–UN = distance from median nerve (medial edge) to ulnar nerve, MN–USP = distance from median nerve (medial edge) to ulnar styloid process, MNW = median nerve width.

* : Student *t* test.

† Mann–Whitney *U* test.

*P* <.05.

The results of the paired *t* test evaluating right–left forearm parameters without considering gender showed no statistically significant differences in any morphometric parameter (*P* > .05, Table 2). Again, no statistically significant differences were observed between the gender groups in the right–left comparison for any of the parameters, for both males and females (*P* > .05, Table 2).

**Table 2:** Sex- and side-based distribution of morphometric parameters of the median nerve and adjacent structures at the distal forearm.

	Female			Male			Total*		
	R (n = 4)	L (n = 4)	*P*	R (n = 10)	L (n = 10)	*P*	R (n = 14)	L (n = 14)	*P*
BL (mm)	47.14 ± 4.22 40.42–53.85†	47.24 ± 0.73 46.08–48.40†	.715‡	48.20 ± 4.00 45.34–51.06†	49.21 ± 6.04 44.89–53.52†	.550§	47.90 ± 3.93 45.63–50.16†	48.64 ± 5.12 45.69–5.60†	.562§
MNL (mm)	34.29 ± 4.96 26.40–42.17†	31.45 ± 6.51 21.09–41.81†	.273‡	39.34 ± 8.70 33.12–45.56†	36.17 ± 12.76 27.05–45.30†	.322§	37.90 ± 7.98 33.29–42.50†	34.82 ± 11.29 28.31–41.34†	.184§
MNW (mm)	9.05 ± 4.24 2.30–15.79†	8.67 ± 0.83 7.35–9.98†	.715‡	7.19 ± 2.09 5.70–8.69†	7.26 ± 1.77 5.99–8.52†	.937§	7.72 ± 2.82 6.07–9.35†	7.66 ± 1.67 6.70–8.62†	.938§
MN–FCR (mm)	3.40 ± 0.67 2.33–4.47†	4.00 ± 0.75 2.80–5.19†	.197‡	3.72 ± 0.94 3.05–4.39†	3.83 ± 0.86 3.21–4.44†	.726§	3.63 ± 0.86 3.13–4.13†	3.88 ± 0.80 3.41–4.34†	.320§
MN–UN (mm)	10.58 ± 2.74 6.22–14.94†	10.37 ± 3.08 5.46–15.27†	>.999‡	12.45 ± 3.97 9.61–15.29†	13.48 ± 4.09 10.55–16.41†	.456§	11.91 ± 3.66 9.80–14.03†	12.59 ± 3.99 10.29–14.89†	.514§
MN–USP (mm)	16.58 ± 2.81 12.10–21.06†	16.94 ± 4.32 10.07–23.81†	>.999‡	18.29 ± 4.41 15.13–21.44†	22.32 ± 5.54 18.35–26.28†	.510§	17.80 ± 3.99 15.49–20.10†	20.78 ± 5.65 17.52–24.04†	.760§
MN–RSP (mm)	21.79 ± 2.30 18.12–25.45†	21.84 ± 3.55 16.19–27.49†	>.999‡	22.49 ± 2.47 20.72–24.25†	19.75 ± 2.94 17.64–21.85†	.950§	22.29 ± 2.36 20.93–23.65†	20.35 ± 3.14 18.53–22.16†	.113§
MNA (cm^2^)	1.80 ± 0.68 0.71–2.88†	1.55 ± 0.66 0.49–2.60†	>.999‡	1.63 ± 0.75 1.09–2.17†	1.29 ± 0.53 0.91–1.66†	0.106§	1.68 ± 0.71 1.27–2.09†	1.36 ± 0.56 1.04–1.68†	0.126§

Descriptive statistics are presented as mean ± standard deviation.

BL = Bistyloid line length, MNA = Median nerve area, MN–FCR = Distance from median nerve (lateral edge) to FCR tendon, MNL = Median nerve length, MN–RSP = Distance from median nerve (lateral edge) to radial styloid process, MN–UN = Distance from median nerve (medial edge) to ulnar nerve, MN–USP = Distance from median nerve (medial edge) to ulnar styloid process, MNW = Median nerve width.

* Paired-samples *t* test was applied. Fourteen cadavers with bilateral upper limbs were analyzed, while 3 unilateral specimens were excluded.

† Indicates the 95% confidence interval.

‡ Wilcoxon signed-rank test.

§ Paired *t* test.

*P* < .05 was considered statistically significant.

To minimize the effects of individual differences and more accurately assess relationships between measurements, the study examined proportional values of specific parameters by gender and side. No statistically significant difference was observed between males and females regarding bistyloid width/MN width, MN area/MN width, and the radial-ulnar styloid ratio of the MN (*P* > .05). In the comparisons between the right and left arms, regardless of gender, a statistically significant difference was observed in the MN area/MN width (*P* = .044) and in the ratio of the distance from the MN to the radial and ulnar styloid processes (*P* = .049). On the other hand, there was no statistically significant difference in the bistyloid width to MN width ratio (*P* = .918).

The relationships between MN parameters and neighboring morphometric variables were assessed using Pearson correlation analysis. A significant positive correlation was observed between BL and the MN width (*R* = 0.693; *P* = .006) and its area (*r* = 0.654; *P* = .011). Median nerve length also demonstrated a strong positive correlation with the MN area (*r* = 0.713; *P* = .004). The strongest positive correlation was found between MN width and area (*r* = 0.779; *P* = .001). A negative correlation was identified between the distances of the MN to the ulnar and radial styloid processes (*r* = −0.703; *P* = .005).

## 4. Discussion

Since the MN becomes superficial in the distal forearm and is often involved in different interventional procedures, morphometric data at this level are important for clinical purposes. This study carefully examined the distances between the MN and anatomical bony landmarks, tendon and neighboring nerve in the distal forearm, measured the MN area, and calculated the ratios between these parameters on cadavers.

The literature emphasizes that the nerve should be evaluated based on fixed reference points to prevent injuries during surgical procedures.^[2,11,12]^ Although the distal wrist line is often used in the clinic, it has been noted to provide a more reliable method for determining the surgical incision line using bone landmarks when the distal wrist line cannot be chosen or soft tissues cannot be assessed by palpation due to trauma-related edema.^[13]^ In this study, the BL measurement between the ulnar and radial styloid prominences in the distal forearm was 1st evaluated, and no significant difference was found between the right and left sides (*P* > .05). The findings of Menge et al,^[14]^ which show no difference in wrist width between the right and left sides, are also consistent with our study. These findings suggest that BL measurement may serve as a consistent anatomical reference for comparative assessments.

In the proximal part of the forearm, the MN runs deep to the FCR, PL, and FDS muscles, and superficial to the flexor digitorum profundus muscle until it reaches the styloid process.^[15]^ Meanwhile, about 5 cm proximal to the distal wrist line, the palmar cutaneous branch separates from the MN.^[16]^ Once the MN gives off this branch, it becomes superficial at the lateral edge of the FDS tendon and extends to the BL. In cases where PL is not present during its course, it is covered only by skin and superficial fascia. In this study, it was found to be 35.79 ± 9.89 mm. At the wrist level, it extends deep into the flexor retinaculum, enters the carpal tunnel, and reaches the palmar surface of the hand. This anatomical location makes the MN one of the most crucial structures in CTS and wrist surgeries. The nerve travels along the bistyloid line throughout its course, reaching a superficial position. In this study, the width of the MN on the BL was found to be 7.65 ± 2.16 mm. Similarly, Brooks et al^[8]^ found the MN width to be 7.85 ± 1.8 mm. Ajayi et al^[5]^ found the MN width to be 5.93 ± (2.51–8) mm below the flexor retinaculum.

In this study, when all forearms were evaluated together, no statistically significant difference was found in the distances of the MN to the bone landmarks RSP and USP. Afroze et al searched for the RSP–MN distance but did not specify the measurement values, however they reported the ratios with forearm parameters.^[11]^ In the study by Ajayi et al,^[5]^ the distances of the MN to the bone landmarks were also seen to be in similar ranges to our study (MN–USP: 24.66 mm, MN–RSP: 22.4 mm). However, since the sample size was limited in both studies and cadavers from different populations were used, the generalization of the obtained data to the general population should be carefully considered. When the distance of the MN to the FCR tendon which is another landmark, was examined, no statistically significant difference was found. In the literature, however, Mizia et al^[17]^ measured the same distance from different planes and found it to be wider (FCR–MN 7.87 mm), and also stated that there was a difference between the right and left arms. Although it is thought that this difference in studies may be due to differences in methodology, further studies with larger sample sizes are needed to obtain stronger and more generalizable results regarding the location of the MN in the distal forearm.

On the other hand, the existence of a palmar cutaneous branch that diverges from the MN at this point and runs along the FCR tendon should also be considered.^[18]^ MacLennan et al^[19]^ stated that during medial injections of FCR, but this method raises the risk of injuring the palmar cutaneous branch. Conversely, it is indicated that slow, controlled needle advancement may help protect this branch. In this context, the measured distance between the MN and the FCR tendon can serve as a supportive anatomical reference, not only for preserving the MN during interventional procedures but also for highlighting the possible course of the palmar cutaneous branch.

In interventional procedures for the forearm, other neurovascular structures in this area may be damaged while attempting to protect the MN.^[20,21]^ For this reason, both the landmarks and their relationships with other neurovascular structures are very important. To achieve this, the distance between the medial edge of the MN and the lateral edge of the ulnar nerve was measured, revealing no difference between the groups. Thus, the distance between the ulnar nerve and the MN across all forearms was measured at 12.09 ± 3.77 mm. In the literature, only studies providing indirect information on the distance between the median and ulnar nerves could be identified.^[16,20,21]^ Therefore, it was stated that to avoid damaging the ulnar nerve, 1 should not go 13 mm medial from the PL muscle.^[21]^ In females, the distance between the median and ulnar nerves (MN–UN) showed a 95% confidence interval of 8.36 to 12.30 mm, which remains below the 13 mm threshold reported in the literature between the PL medial to the MN and the UN. In males, the lower bound of the 95% confidence interval was 11.04 mm, suggesting a closer anatomical relationship between the MN and the UN. Based on these findings, excessive medial deviation from the PL tendon may increase the risk of ulnar nerve injury during interventional procedures; however, this risk should be evaluated in consideration of individual anatomical characteristics, nerve variations, and sex-related morphometric differences.

In this study, we collected not only length measurements but also data on the area occupied by the MN in the forearm. Accordingly, the MN was found to occupy an area of 1.49 ± 0.63 cm^2^ in the distal forearm. In addition to length measurements, characterization of the nerve occupied area at this level may assist in anatomical orientation and support timely decision-making during trauma management, surgical planning, and interventional procedures. In the existing literature, the positional relationships of the MN in the distal forearm have predominantly been evaluated using linear distance measurements, and no study has specifically examined the area occupied by the nerve at this level.^[22]^ By providing these morphometric data, the present study adds supplementary anatomical reference information to the existing literature Furthermore, these findings may provide anatomical reference data for future studies in which MN area is evaluated within clinical and radiological imaging contexts.

To minimize the effect of individual variability, proportional values were calculated, and no statistically significant differences were found between sexes. However, when the right and left forearms were compared regardless of sex, statistically significant differences were observed in the MN area/MN width and in the ratio of the distance from the MN to the RSP and USP (*P* < .05). Nevertheless, since the corresponding *P* values were very close to the .05 threshold, these findings should be interpreted with caution. Further studies with larger sample sizes are warranted to confirm the robustness and clinical relevance of these results. Correlation analysis revealed that as the bistyloid length increased, the length and area of the MN also increased. A strong negative correlation was found between the distances of the MN to the USP and RSP (*r* = −0.703, *P* = .005). These results are considered to be a reflection of fundamental spatial geometry and to be confirmatory in nature, rather than presenting new anatomical findings.

**Figure 2.**
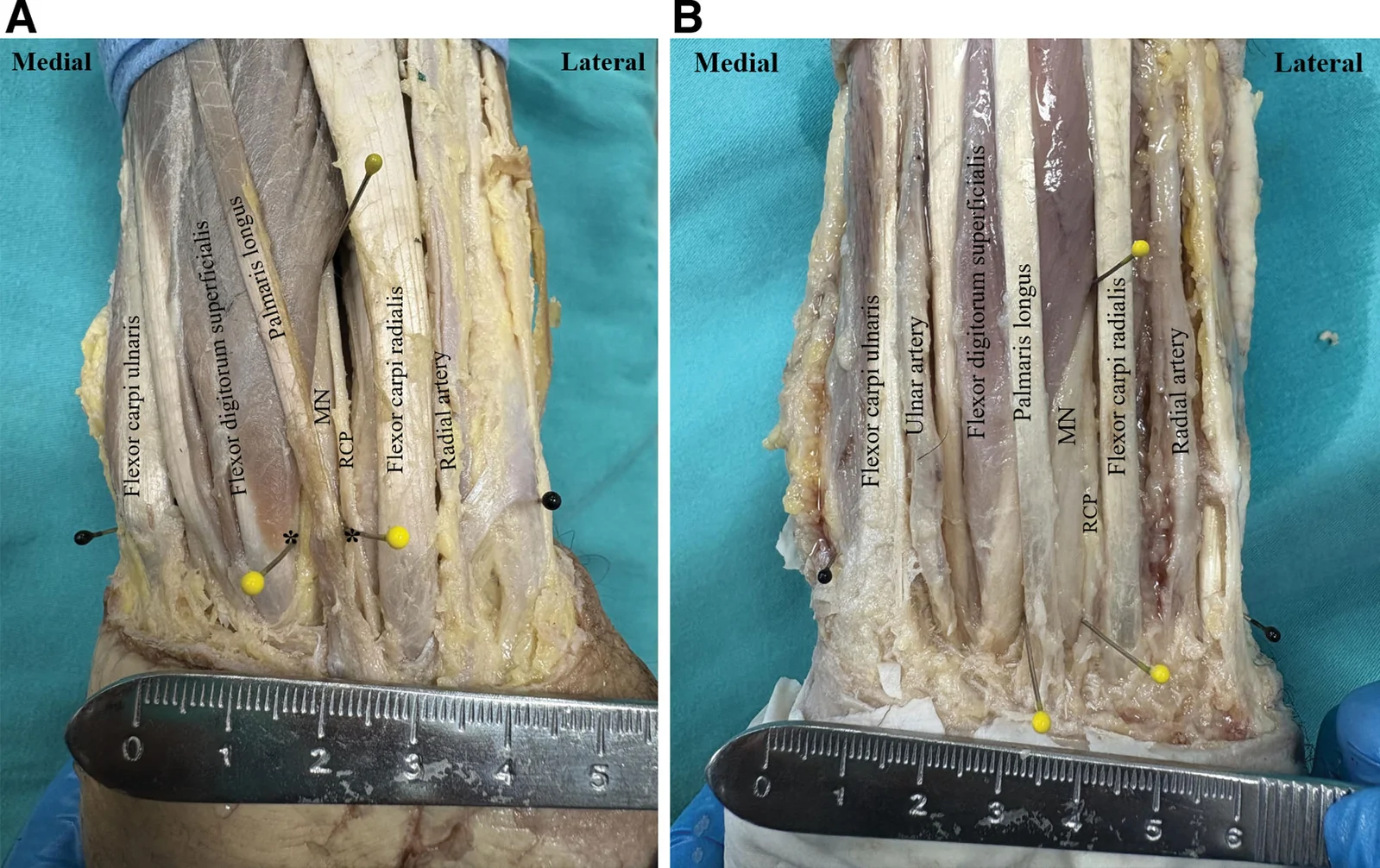
Anterior view of the left distal forearm. Black pins indicate the bistyloid line. The distal yellow pins mark the position of the median nerve on this line, and the proximal yellow pin shows the point where the nerve becomes visible at the lateral edge of the flexor digitorum superficialis. (A) In a 49-year-old male cadaver, the median nerve crosses the medial border of the palmaris longus tendon. (B) In a 90-year-old male cadaver, the median nerve lies deep to the palmaris longus tendon but does not cross its medial border. The palmar cutaneous branch of the median nerve courses along the medial border of the flexor carpi radialis tendon.

In addition to these morphometric observations, the position of the MN with respect to adjacent tendinous structures also warrants consideration when interpreting its distal forearm anatomy. Racasan et al^[21]^ reported that in 88% of cases, the MN was located in the ulnar position relative to the PL tendon, and literature reviews have found studies reaching similar conclusions.^[6,8]^ While Ajayi et al^[5]^ suggest that PL can help locate the MN, Hou et al^[6]^ research shows that the MN position relative to the PL tendon is highly variable. Therefore, using the PL tendon as a reliable anatomical reference point for locating the MN remains a topic of controversy. In addition, this structure cannot be used as an anatomical reference point in approximately 11% of patients with no PL muscle.^[14]^ In this study, the presence of the PL muscle was 58%. It was not used as a separate variable in morphometric analyses due to the low incidence of PL and the destruction of the PL muscle, as some cadavers had been previously opened. In a few cases involving PL, it was observed that the MN extended medially over the PL tendon at the level of the distal wrist line (Fig. 2).

These results and literature review suggest that the placement of the MN relative to the PL tendon can vary significantly among individuals and may not always be a reliable anatomical landmark. Similarly, the literature also shows that other anatomical structures besides the PL have been used in interventional procedures for CTS. Accordingly, the preferred injection sites are shown below.

- Between the PL and the flexor carpi ulnaris^[23]^
- To the medial side of the PL^[24]^
- Between the PL and the FCR^[25]^
- To the medial side of the FCR^[8,19]^
- From inside the FCR tendon^[20,21]^
- 30%–33% of the bistyloid width away from the RSP^[14,20]^

The common feature of these methods is identifying reference points based on specific tendons or superficial anatomical structures to define the safe zone. However, these methods are not equally reliable for all patients due to anatomical differences in the MN and variations in measurements among individuals. In particular, the absence of the PL muscle, the shift of the MN to the radial or ulnar side, and variations in the nerve should be considered when planning interventional procedures. To reduce the risk of iatrogenic injury during blind injections that may arise from these variations, the use of radiological imaging techniques, such as ultrasound guidance, may be more advantageous.

### 4.1. Limitations

This study has several limitations. As the data were obtained from cadaveric specimens, the findings cannot be directly extrapolated to living subjects and should be interpreted accordingly. In addition, the relatively small sample size and the imbalance between male and female cadavers may limit the generalizability of the results. Although standardized measurement techniques were used, the absence of formal inter-rater reliability assessment should be considered when interpreting the reproducibility of the measurements. Future studies including reliability analyses and larger, more balanced samples may further strengthen the clinical interpretability of these findings.

## 5. Conclusion

Based on the morphometric features observed in this cadaver-based study, 2 anatomical regions can be evaluated to understand the relative position of the MN at the wrist level: the area extending from the FCR tendon to the lateral edge of the MN, and the area between the lateral border of the ulnar nerve and the medial edge of the MN. These regions aim to provide clinicians with supporting anatomical information through the detailed presentation of distance measurements, rather than defining definitive safe zones. Furthermore, considering the area occupied by the MN during surgical or interventional procedures can contribute to reducing the risk of involuntary nerve injury. However, it should be noted that the data obtained are from cadavers and the number of cadavers should be taken into account; these findings should be supported by surgical and radiological studies in living individuals before being used in clinical practice.

## Acknowledgments

We would really like to thank Assist. Prof Dr Fulden Cantaş Türkiş from the Department of Biostatistics, Faculty of Medicine, Muğla Sitki Koçman University, Muğla/Turkey, for her contribution in reviewing our statistical analyses.

## Author contributions

**Conceptualization:** Ceren Uğuz Gençer, Hüseyin Aydoğmuş, Mehmet İlkay Koşar.

**Data curation:** Ceren Uğuz Gençer, Mustafa Deniz Yörük, Zeynep Nisa Karakoyun.

**Formal analysis:** Ceren Uğuz Gençer, Hüseyin Aydoğmuş.

**Supervision:** Ceren Uğuz Gençer, Hasan Tetiker.

**Validation:** Ceren Uğuz Gençer, Hüseyin Aydoğmuş, Mehmet İlkay Koşar.

**Visualization:** Mustafa Deniz Yörük, Zeynep Nisa Karakoyun.

**Writing – original draft:** Ceren Uğuz Gençer, Hasan Tetiker.

**Writing – review & editing:** Ceren Uğuz Gençer, Hüseyin Aydoğmuş, Mehmet İlkay Koşar, Hasan Tetiker.
